# Unveiling kaposi sarcoma viral antigens

**DOI:** 10.18632/oncotarget.19106

**Published:** 2017-07-08

**Authors:** Jaeyeun Lee, John G. Kosowicz, Richard F. Ambinder

**Affiliations:** Department of Oncology, Johns Hopkins School of Medicine, Baltimore, MD, USA

**Keywords:** Kaposi sarcoma, lenalidomide, pomalidomide, MHC-I, immunomodulatory drugs, KSHV

Kaposi sarcoma (KS) is a debilitating, disfiguring and often fatal disease. The tumor is always associated with Kaposi sarcoma herpesvirus (KSHV, also known as HHV8) [1]. It is often, perhaps always, associated with immune deficiency. In transplant recipients KS is associated with pharmacologic immunosuppression, while in HIV patients KS is associated with low CD4 T cell counts. Improvement in immune function is often associated with KS regression. In transplant recipients immune function improves following withdrawal or change in the immunosuppressive regimen. In HIV patients immune function improves with antiretroviral therapy. Lenalidomide and pomalidomide both have activity in the treatment of KS [2]. In this issue of Oncotarget, Davis et al present evidence suggesting that efficacy may relate to immunomodulatory effects [3].

The possibility that the antineoplastic properties of thalidomide, lenalidomide and pomalidomide in part reflect immune modulation will come as no surprise to those who have been following the immunomodulatory drug (IMiD) saga [4]. In the early 1960s, thalidomide was associated with severe fetal limb deformities. Subsequently, thalidomide showed efficacy in treating a complication of leprosy as well as in treating multiple myeloma. Congeners lenalidomide and pomalidomide were synthesized and shown to be more effective in treatment of multiple myeloma and less toxic. Immunomodulatory properties were recognized in T cells, T regulatory cells (Tregs), natural killer (NK) cells. The IMiDs bind to cereblon, the substrate receptor for an E3 ligase complex, leading to modulation of the substrate specificity of the E3 ligase activity so as to recognize neosubstrates leading to polyubiquitinylation and degradation. The substrates include Ikaros and Aiolos which are repressors of T cell function and thus help to explain T cell activation. However, the present investigation is not focused on the effector arm of the immune system but on the tumor cells harboring KSHV.

KSHV infection down regulates MHC-I and ICAM/B7 expression protecting infected cells from CD8^+^ T and NK cells, respectively [5, 6]. Davis et al demonstrate that lenalidomide and pomalidomide restore MHC-I expression during viral lytic activation and ICAM/B7-2 expression in latently infected KSHV (+) cancer cells. MHC-I down regulation is mediated by K3 and K5, two KSHV-encoded E3 ubiquitin ligases. Davis et al demonstrate that pomalidomide specifically blocks the effects of the viral protein K3 on MHC-I down regulation—but the mechanism isn’t clear [3]. Their findings raise the possibility that pomalidomide directly interacts with K3 in parallel with cereblon. Alternatively, there may be complex interactions among the E3-ligase complexes that remain to be elucidated.

Whatever the mechanism by which pomalidomide blocks viral functions that functionally veil KSHV cells from immune surveillance, it seems likely that this “unveiling” helps explain the impressive clinical responses reported with pomalidomide. Other advances in immunotherapy make the findings all the more interesting. Checkpoint inhibitors are playing an ever more important role in cancer treatment [7]. Tumors with high mutational density (encoding neoantigens) are among the most sensitive to such therapy. Evidence is also emerging that virus-associated tumors are very sensitive [8]. Trials of checkpoint inhibitors in patients with HIV and cancer including KS are presently ongoing. As Davis et al. mention in the discussion, re-expression of immune cell surface molecules in association with imide treatment may enhance the efficacy of the check point inhibitors. Combinations of IMID and checkpoint inhibitors are already ongoing in multiple myeloma. Viral associated tumors are an attractive next venue.
